# Fuzzy context specific matched molecular pairs

**DOI:** 10.1186/1758-2946-6-S1-P44

**Published:** 2014-03-11

**Authors:** Peter Schmidtke, Vincent le Guilloux

**Affiliations:** 1 Discngine SAS, Paris, 75011, France

## 

Matched molecular pairs (MMPs) are commonly used to assess the importance of chemical modifications on small molecules versus a particular property [1]. One major advantage of MMPs is the direct interpretation by medicinal chemists. Despite the popularity of MMPs several drawbacks hinder their systematic applications in drug discovery projects. First, in order to derive sufficiently representative statistics for extracting design rules rather large datasets of molecules and activities are needed. Second, MMPs are often used without context specificity, likely because of the lack of sufficient data. This results in weak statistics. One transformation could result to be beneficial, but is detrimental in reality once the context taken into account [2]. Context specific MMP analysis is clearly advantageous for in depth understanding of SAR, but hindered due to lack of data.

Here we present a novel methodology for providing robust statistics for fuzzy context specific MMPs even on medium sized data-sets. Molecules are transformed to a reduced graph using the Discngine Pharmacophore Graph methodology [3], a molecular graph reduction method closely related to classical reduced graphs [4]. Next, molecular fragmentation and MMP detection is directly performed on the pharmacophore graph. The herein used pharmacophore graph representation allows to group together very similar contexts and/or fragments and thus increase population size compared to classical MMP analysis.

Validation of the fuzzy context specific MMP (fcsMMP) is presented and outcome is compared to classical MMP analysis. Last the Discngine Network framework is used to organize the derived design rules for efficient large scale mining and results extraction in a real world Med Chem context and applications are shown.
